# Health Inequality Among the Elderly in Rural China and Influencing Factors: Evidence from the Chinese Longitudinal Healthy Longevity Survey

**DOI:** 10.3390/ijerph16204018

**Published:** 2019-10-20

**Authors:** Changjian Pan, Qiuyan Fan, Jing Yang, Dasong Deng

**Affiliations:** 1 Economics and Management School, Wuhan University, Wuhan 430072, China; panchangjian@whu.edu.cn; 2 Centre for Social Security Studies, Wuhan University, Wuhan 430072, China; dengdasong@vip.sina.com

**Keywords:** health inequality, elderly in rural China, influencing factors

## Abstract

Based on data from the Chinese Longitudinal Healthy Longevity Survey (CLHLS), this paper calculates the health distribution of the elderly using the Quality of Well-Being Scale (QWB) score, and then estimates health inequality among the elderly in rural China using the Wagstaff index (WI) and Erreygers index (EI). Following this, it compares health inequalities among the elderly in different age groups, and finally, uses the Shapley and recentered influence function-index-ordinary least squares (RIF-I-OLS) model to decompose the effect of four factors on health inequality among the elderly in rural China. The QWB score distribution shows that the health of the elderly in rural China improved with social economic development and medical reform from 2002 to 2014. However, at the same time, we were surprised to find that the health level of the 65–74 years old group has been declining steadily since 2008. This phenomenon implies that the incidence of chronic diseases is moving towards the younger elderly. The WI and EI show that there is indeed pro-rich health inequality among the rural elderly, the health inequality of the younger age groups is more serious than that of the older age groups, and the former incidence of health inequality is higher. Health inequality in the age group of 65–74 years old is higher than that in other groups, and the trend of change fluctuated downward from 2002 to 2014. Health inequality in the age group of 75–84 years old is lower than that in the group of 65–74 years old, but higher than that in the other age groups. The results of Shapley decomposition show that demographic characteristics, socioeconomic status (SES), health care access, and quality of later life contributed 0.0054, 0.0130, 0.0442, and 0.0218 to the health inequality index of the elderly, which accounted for 6.40%, 15.39%, 52.41%, and 25.80% of health inequality index. From the results of RIF-I-OLS decomposition, this paper has analyzed detailed factors’ marginal effects on health inequality from four dimensions, which indicates that the health inequality among the elderly in rural China was mainly caused by the disparity of income, medical expenses, and living arrangement.

## 1. Introduction

With rapid aging of the population, the problem of health inequality among the elderly poses a severe challenge to the improvement and construction of health care systems and pension service systems in various countries. Health inequality among the elderly appears not only in developed countries but also in developing countries. Nearly 60% of people with dementia live in low- and middle-income countries, and this proportion is expected to increase rapidly during the next decade, which may contribute to increasing inequalities among countries and populations [1]. China is facing serious population aging issues because of unintended consequences of the economic reforms and social policies which began in the 1980s. Despite the remarkable progress in health status (e.g., the increasing life expectancy) in China, there remain large inequalities across the country, especially in rural areas. Although the overall health condition of rural residents has gradually increased due to continuous improvement of the New Cooperative Medical Schemes (NCMS), the health inequality of the elderly in rural China deserves attention. Influenced by the rapid aging and huge migration out of rural labor, the problems of health inequity among the rural elderly are becoming more and more difficult to deal with.

Health inequalities can be attributed to biological variations, but also other variables, including demographic, socioeconomic, and medical conditions, etc. In view of the influencing factors of health inequality among the elderly, scholars have conducted many useful studies and explorations. Some studies have found that urban residents are more likely to suffer from non-communicable diseases, and that urbanization had been proven to impose a penalty on perceived health in China [2,3]. Other studies have shown that the health inequality of the elderly in rural areas deserves more attention, because the rural elderly are more likely to experience functional limitations than the urban elderly and less likely to survive a two-year follow-up period [4]. Specifically, factors affecting health inequality include age, gender, income, and education [5]. Growing evidence indicates that a lower socioeconomic status (SES) is associated with poorer health, and that health inequalities favor high-income groups [6]. Some scholars have found that the poor usually have less access to health care than the better-off, despite having higher levels of need. Notwithstanding their lower levels of utilization, the poor often spend more on health care as a share of their income than the better-off [7,8,9]. However, Nan found that higher income inequality did not have an impact on the likelihood of having health problems in China [10]. In addition, education also significantly impacts health inequality of the elderly, and the impact of education on health inequalities is non-linear. Chen and Steven employed two cross-sections to estimate and decompose the income-related inequality in self-assessed health status. The increased level of income outweighed the increased inequality in income, and disparities in access to education contributed to increases in inequality [11]. Liu concluded that less education, rural residence, unemployment, and a lower income were associated with functional disability in Chinese older adults [12]. The launch of the New Rural Cooperative Medical Scheme (NRCMS) in 2003 was designed to reduce inequity in access to health care for the rural population. Although these reforms have offered some relief to the poor, concerns remain [13]. Cai proved that health insurance had the opposite effects on health inequality for urban and rural populations, resulting in lower inequality for urban populations and higher inequality for their rural counterparts [14].

The literature review shows that the existing research has the following shortcomings. Firstly, the research on the health inequality of rural elderly and its influencing factors usually uses the concentration index to analyze the differences in residents’ health, but it has obvious defects. Secondly, research studies based on an empirical analysis of health impact factors are great in number, but empirical research specifically aimed at health inequality of the elderly is not adequate. Although previous studies have explored determinants of the elderly’s health in China, few published studies have investigated the health inequality of rural samples. Studies focusing on the evolutionary determinants of rural elderly health inequality are still limited. Thirdly, the existing research on factors affecting the health inequality of the elderly mainly focus on the socioeconomic status (SES) dimension, which is closely related to the health level and quality of life of the elderly, such as medical services and pension dimensions, and is not deep enough, so the conclusions are limited to some extent.

**Figure 1:**
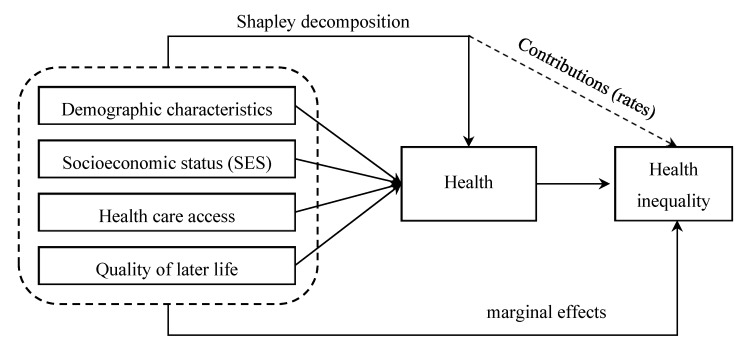
Core research framework.

This paper uses the 2002–2014 Chinese Longitudinal Healthy Longevity Survey (CLHLS) to investigate the health level of the elderly in China, describes the health inequalities and trends of the rural elderly, and uses the Shapley decomposition method and recentered influence function-index-ordinary least squares (RIF-I-OLS) model to decompose the influencing factors of rural elderly health inequality in order to provide an empirical reference for promoting the equal and healthy aging of rural elderly. The core research framework of this paper is shown in Figure 1. In this figure, demographic characteristics, socioeconomic status (SES), health care access, and quality of later life can lead to health inequalities among the elderly by affecting their health. These effects will be analyzed and discussed in detail in this paper. Compared with the existing research, the possible innovations of this paper are as follows: this paper uses the multi-phase data of CLHLS (2002–2014) for measuring the health inequality of the rural Chinese; by using the decomposition methods of Shapley and RIF-I-OLS, this paper tries to analyze the effects of demographic characteristics, socioeconomic status (SES), health care access, and quality of later life on health inequality among the elderly in rural China. This study is conducive to enriching the theoretical discussion of health inequality, and it is also a useful supplement to the existing studies.

## 2. Materials and Methods

### 2.1. Data

The main data were obtained from the Chinese Longitudinal Healthy Longevity Survey (CLHLS), which is organized by the Center for Healthy Ageing and Development and the National Development Research Institute of Peking University. The survey covers 22 provinces, autonomous regions, and municipalities (Beijing, Tianjin, Hebei, Shanxi, Liaoning, Jilin, Heilongjiang, Shanghai, Jiangsu, Zhejiang, Anhui, Fujian, Jiangxi, Shandong, Henan, Hubei, Hunan, Guangdong, Guangxi, Chongqing, Sichuan, Shaanxi, and Hainan). This paper reports on the data from seven follow-up surveys from 1998, 2000, 2002, 2005, 2008, 2011, and 2014, respectively. The population of the surveyed area accounts for about 85% of the country’s total population, and the representative advantage of the sample is obvious. The initial survey was for elderly people aged 80 years and older. Since 2002, the survey has expanded to include elderly people aged 65 and over. According to the time nodes and data age coverage of China’s entry into the aging society, this paper selects the survey samples collected since 2002 and extracts rural sub-samples for analysis. The pooled cross-section data processed from the 2002–2014 survey year data excluded missing data and outliers, and 19,341 rural elderly sample sets of data were obtained. At the same time, according to the research needs, some macro data collected by the Economy Prediction System (EPS), can be found at http://olap.epsnet.com.cn/, are embedded, including demographic, economic, and medical statistics. The data set structure is shown in Table 1 below.

### 2.2. Methods

#### 2.2.1. Health Measures

In view of the main analysis of the health inequalities of the elderly, it is necessary to compare the health status of different elderly individuals. Therefore, the Quality of Well-Being Scale (QWB) was selected as the measure of health of the elderly. This is also in line with the World Health Organization’s reference to “improving the quality of life of the elderly”. The QWB is compiled by Kaplan and Anderson (1988) and is commonly used to describe health-related quality of life (HRQOL) for larger groups or samples and to provide information for epidemiological studies and public health policies [15]. The value of QWB is between 0 and 1. The closer the value is to 0, the worse the health condition (or means death) is, and the closer the value is to 1, the better the health (complete health) is. The QWB health score is calculated as follows:

(1) QWB=1+MOB+PAC+SAC+CPX.

In the formula, MOB (mobility scale, MOB), PAC (physical activity scale, PAC), SAC (social activity scale, SAC), and CPX (symptom/problem complexes, CPX), respectively, represent the weights corresponding to the measured items of the four dimensions of the action indicator, the physical activity index, the social activity indicator, and the symptom indicator. These weights and the corresponding number of the CLHLS questionnaire are shown in Table 2. Equation (1) can be used to calculate the health score of each person.

#### 2.2.2. Measurement of Health Inequality

When measuring the difference in health among the elderly, sociologists and economists are interested in exploring the health inequalities related to socioeconomic status (SES) (usually represented by income), so different forms of multiple health inequalities have been developed. Among them, the Wagstaff index (*WI*) and Erreygers index (*EI*) were respectively proposed by Wagstaff [16] and Erreygers [17], and they are widely used in the research field of health inequality [14]. Both of these indices are based on a bi-variate approach which examines both health distributions and income distributions. The specific calculation formulas of *WI* and *EI* are as follows:

(2) $$WI\left(h|y\right)=\frac{1}{n}\sum_{i=1}^{n}\left[\frac{\left(h_{max}-h_{min}\right)h_{i}}{\left(h_{max}-\bar{h}\right)\left(\bar{h}-h_{min}\right)}\left(2R_{i}-1\right)\right],$$

(3) $$EI\left(h|y\right)=\frac{1}{n}\sum_{i=1}^{n}\left[\frac{4h_{i}}{\left(h_{max}-h_{min}\right)}\left(2R_{i}-1\right)\right],$$

where, h is the health score of the elderly; $\bar{h}$ represents the average health level of the elderly; y is the per capita income of the elderly’s families; and *R_i_* is the rank of the per capita household income in the total sample. The values of *WI* and *EI* are between −1 and 1. When the *WI* and *EI* values are between −1 and 0 or 0 and 1, it indicates that there is health inequality in the elderly group with a low (or high) income. The greater the absolute value of *WI* and *EI* is, the deeper the health inequality of the rural elderly is.

#### 2.2.3. Decomposition Methods of Factors Influencing Health Inequality

Firstly, the Shapley decomposition method [18] was used to measure the contribution and contribution rate of the four dimensions in terms of health inequality among the rural elderly in China. The Shapley decomposition method is divided into two steps: The first step is to design the regression equation of the determinants of the health of the elderly, to estimate the impact of different factors on the health of the elderly, and the second step is to measure the health inequality among the elderly. Indicators were allocated to both sides of the equation to measure the contribution of different influencing factors to the health inequality among the elderly. The basic regression equation designed in this paper is Equation (4):

(4) $$H=a+\beta_{1}DC_{1}+\beta_{2}SES_{2}+\beta_{3}HCA_{3}+\beta_{4}QLL_{4}+\varepsilon .$$

Here, *H* indicates the health level of the elderly; *α* represents the intercept term of the model; *DC, SES, HCA*, and *QLL* indicate the demographic characteristics, socioeconomic status (SES), health care access, and quality of later life respectively; *β1–β4* are vector sets of the parameters to be estimated, and ε represents random perturbation vector groups. Based on the regression equation, this paper uses the contribution Shapley2 of the stata 15.0 software to decompose the four factors’ contribution (rate) to health inequality among the elderly in rural China.

Secondly, this paper uses the RIF-I-OLS model to analyze the marginal effects of the specific factors included in the four dimensions on elderly health inequality. The process of the RIF-I-OLS method is to use the recentered influence function (*RIF*) estimates of the health inequality index to find the relationship between the *RIF* and the explanatory variables, and establish the regression function between the health inequality index and the explanatory variables to achieve causal recognition. The method is mainly divided into two steps. The first step is to estimate the *RIF* value of the target concentration index (such as *WI*, *EI*, etc.), and the second step is to use the *RIF* estimation value of the health inequality index as the explanatory variable and various factors *X* as explanatory variables to implement the ordinary least squares (OLS) regression process. The derivation process of the model is referred to the paper of Heckley et al. [19] or the research of Cai et al. [14]. The specific process is as follows:

**Step 1.** Obtain the recentered influence function (*RIF*) of the binary rank dependent index (I). 

The *RIF* is derived from the influence function *(IF),* which originates from the statistical robustness literature. Hampel introduced the concept of *IF* to explore how various statistics are interfered with or affected by specific observations [20]. However, the *RIF* has the same properties as the *IF*, except for the different expected values and decomposition methods [21,22]. In this paper, we first introduce the concepts of *IF* and *RIF* in a uni-variate setting, and then derive the *RIF* of the general binary rank-dependent index.

The health inequality index *I* is a function of the joint probability distribution $F_{H,F_{Y}}$. Let $G_{h,F_{Y}\left(y\right)}$ be a binary distribution function, which can be obtained when the noise of the joint distribution h of $F_{Y}\left(y\right)$ and $F_{H,F_{Y}}$ is infinitely small. The expression for $G_{h,F_{Y}\left(y\right)}$ is:

(5) $$G_{h,F_{Y}\left(y\right)}=\left(1-\tau \right)F_{H,F_{Y}}+\tau \delta_{h,F_{Y}\left(y\right)},$$

where $\delta_{h,F_{Y}\left(y\right)}$ represents the joint cumulative distribution function of the joint probability measure, and its expression is:

(6) $$\delta_{h,F_{Y}\left(y\right)}\left(l,r\right)=\left\{ \begin{array}{l} 0,l<h\cup r<F_{Y}\left(y\right)\\ 1,l\geq h\cap r>F_{Y}\left(y\right) \end{array}\right..$$

By plotting l and r with H and $F_{Y}$, respectively, we can get the influence function for the binary level dependence index of H and $F_{Y}$ as follows:

(7) $$IF\left(h,F_{Y}\left(y\right);v^{I}\right)=\frac{\partial v^{I}\left(G_{h,F_{Y}\left(y\right)}\right)}{\partial \tau }{|}_{\tau =0}=\lim_{\tau \to 0}\frac{v^{I}\left(G_{h,F_{Y}\left(y\right)}\right)-v^{I}\left(F_{H,F_{Y}}\right)}{\tau }.$$

According to the definition of *RIF*, the re-centralization influence function *RIF* of the binary rank dependence index is obtained:

(8) $$RIF\left(h,F_{Y}\left(y\right);v^{I}\right)=v^{I}\left(F_{H},F_{Y}\right)+IF\left(h,F_{Y}\left(y\right);v^{I}\right).$$

When the expectation of *IF* is zero, the expectation of *RIF* is equal to the original distribution statistic. This is a useful attribute as the standard regression tool for mean values is allowed to be applied to multiple statistics and thus decomposed. If the absolute concentration index function is $v^{AC}$, the *RIF* of the two indices of *WI* and *EI* can be expressed as Equations (9) and (10), respectively:

(9) $$RIF\left(h,F_{Y}\left(y\right);v^{WI}\right)=v^{WI}\left(F_{H},F_{Y}\right)+\frac{-\left(b_{H}-a_{H}\right)\left[\left(b_{H}+a_{H}-2\mu_{H}\right)\left(h-\mu_{H}\right)\right]}{{\left(\left(b_{H}-\mu_{H}\right)\left(\mu_{H}-a_{H}\right)\right)}^{2}}\cdot v^{ACI}\left(F_{H},F_{Y}\right)+\frac{b_{H}-a_{H}}{\left(b_{H}-\mu_{H}\right)\left(\mu_{H}-a_{H}\right)}IF\left(h,F_{Y}\left(y\right);v^{ACI}\right)$$

(10) $$RIF\left(h,F_{Y}\left(y\right);v^{EI}\right)=v^{EI}\left(F_{H},F_{Y}\right)+\frac{4}{b_{H}-a_{H}}IF\left(h,F_{Y}\left(y\right);v^{ACI}\right).$$

In the above formulas, $a_{H}$, $b_{H}$, and $\mu_{H}$ representative the upper bound, lower bound, and average of health level respectively.

**Step 2**. Construct the RIF-I-OLS model by *RIF* regression decomposition. Using *RIF* regression, two parameters can be estimated: one is the marginal effects of the covariate *X* on the function, which is an individual effect, and the other is the unconditional quantile effect, that is, the population effect. According to the definitions of *IF* and *RIF* mentioned above, $v^{I}\left(F_{H},F_{Y}\right)$ can be expressed as the expected value of *RIF*, namely,

(11) $$v^{I}\left(F_{H},F_{Y}\right)=\int_{-\infty }^{\infty }RIF\left(h,F_{Y}\left(y\right);v^{I}\right)\cdot dF_{H,F_{Y}}\left(h,F_{Y}\left(y\right)\right)=E\left[RIF\left(H,F_{Y};v^{I}\right)\right].$$

In order for v to be associated with covariate $v^{I}\left(F_{H},F_{Y}\right)$, the method of Firpo et al. (2009) [21] should be followed to apply the iterative expectation method to convert $v^{I}\left(F_{H},F_{Y}\right)$ to conditional expectation:

(12) $$v^{I}\left(F_{H},F_{Y}\right)=\int_{-\infty }^{\infty }RIF\left(h,F_{Y}\left(y\right);v^{I}\right)\cdot dF_{H,F_{Y}}\left(h,F_{Y}\left(y\right)\right)=\int_{-\infty }^{\infty }E\left[RIF\left(H,F_{Y};v^{I}\right)|X=x\right]\cdot dF_{X}\left(x\right),$$

where $F_{X}$ is the cumulative distribution function (CDF) of *X*. In this way, the decomposition of $v^{I}\left(F_{H,F_{Y}}\right)$ comes down to the problem of estimating the conditional expectation, which can be solved by the standard regression method. If λ(X,ε) represents the general function of covariate *X* and error ε, the conditional expectation of $RIF\left(h,F_{Y}\left(y\right);v^{I}\right)$ can be modeled as:

(13) $$E\left[RIF\left(H,F_{Y};v^{I}\right)|X=x\right]=\lambda \left(X,\varepsilon \right).$$

Therefore, the marginal effects of the covariate *X* on the function can be given by the partial derivative of the conditional period of $RIF\left(h,F_{Y}\left(y\right);v^{I}\right)$, namely,

(14) $$\frac{dE\left[RIF\left(H,F_{Y};v^{I}\right)|X=x\right]}{dx}=\frac{d\lambda \left(X,\varepsilon \right)}{dx}.$$

The unconditional quantile effect is a vector of the average partial derivatives, denoted by $\gamma \left(v^{I}\right)$, and its expression is:

(15) $$\gamma \left(v^{I}\right)=\int_{-\infty }^{\infty }\frac{dE\left[RIF\left(H,F_{Y};v^{I}\right)|X=x\right]}{dx}\cdot dF_{X}\left(x\right)=\int_{-\infty }^{\infty }\frac{dE\left(X,\varepsilon \right)}{dx}\cdot dF_{X}\left(x\right).$$

Assuming that λ(⋅) is a linear function, the ordinary least squares (OLS) method is used for parameter estimation, and the RIF-I-OLS estimation model can be obtained. The advantages of RIF-I-OLS in terms of operation and interpretation are obvious. It allows flexible functional forms such as nonlinear or high-order conversion, as in the case of standard OLS, and the estimation results are easy to understand.

When the coefficients of the error term and the covariate are μ and ψ, under the assumption of a linear positive error term and a zero condition mean, then Equations (13)–(15) can be written as Equations (16)–(18):

(16) $$E\left[RIF\left(H,F_{Y};v^{I}\right)|X=x\right]=X^{\prime }\psi +\mu ,$$

(17) $$\frac{dE\left[RIF\left(H,F_{Y};v^{I}\right)|X=x\right]}{dx}=\frac{d\left[X^{\prime }\psi +\mu \right]}{dx}=\psi ,$$

(18) $$\gamma \left(v^{I}\right)=\int_{-\infty }^{\infty }\frac{d\left[X^{\prime }\psi +\mu \right]}{dx}\cdot dF\left(x\right)=\psi .$$

Under the assumption of a linear and zero conditional mean, the marginal effect and the unconditional partial effect are the same. Therefore, the OLS optimal estimation method is used for *RIF* regression.

### 2.3. Variables

#### 2.3.1. Interpreted Variables

In this paper, the interpreted variables of Shapley decomposition and RIF-I-OLS decomposition are the health score and health inequality index. The health score is measured by the QWB scale, and the health inequality index is characterized by *WI* and *EI*.

#### 2.3.2. Explanatory Variables

Explanatory variables of this paper include the four dimensions of demographic characteristics, socioeconomic status (SES), health care access, and quality of later life. Demographic characteristics include gender, age groups, and marital status; socioeconomic status (SES) includes income inequality, education, and region; health care access includes timely medical treatment, medical facilities, medical expenses, and the main medical payment channel; and quality of later life includes living arrangements, pension, and physical exercise. A statistical description of the main variables is given in Table 3.

## 3. Results

In order to deeply understand the health inequality of the elderly and analyze the influencing factors and their influence mechanism, this paper calculates the health distribution of the elderly by using the QWB score, and then estimates the health inequality of the elderly in rural China by using the Wagstaff index (WI) and Erreygers index (EI). After this, it compares health inequalities among the elderly in different age groups, and finally, uses the Shapley and RIF-I-OLS model to decompose the four dimensions affecting health inequality among the elderly in rural China.

### 3.1. Health Characteristics of the Elderly in Rural China

As shown in Table 4, the health QWB score in the group of 65–74 years old firstly rises to 0.7832 (2008) from 0.7730 (2002), and then descends to 0.7770 (2014). The QWB score in the group of 75–84 years old continues to rise to 0.7276 (2014) from 0.7128 (2002). Both the QWB values in the groups of 85–94 years old and above 95 years old rise from 2002 to 2014. Additionally, the QWB score of the 85–94 years old group rose from 0.6357 (2002) to 0.6600 (2014), and the QWB score of the above 95 years old group rose from 0.5639 (2002) to 0.5868 (2014). From the comparison chart, it can be clearly seen that the health level of the elderly in 2014 had improved compared with 2002, which may be the result of social welfare improvement brought about by social economic development and medical reform. However, at the same time, we also see that the health level of the younger elderly has been declining since 2008, which implies that the incidence of chronic diseases is moving towards the younger elderly.

### 3.2. Health Inequality Among the Elderly in Rural China

**Figure 2:**
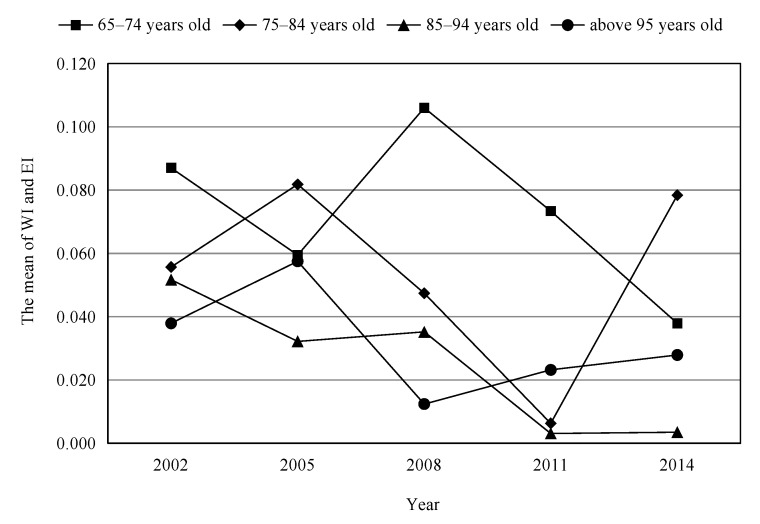
The mean of Wagstaff index (WI) and Erreygers index (EI) of the elderly in different age groups from 2002 to 2014.

Table 5 and Figure 2 show the distribution of *WI* and *EI* for the health inequality index of the elderly among different age groups. These data show that there is indeed pro-rich health inequality among the rural elderly, the health inequality of the younger age groups is more serious than that of the older age groups, and the former incidence of health inequality is higher. The tendency of health inequality among the elderly in rural areas decreased from 2002 to 2014, but it did not disappear, which indicates that socioeconomic development and rural health system reform have improved the health inequality of the rural elderly. This improvement effect is positive, but does not completely eliminate the health inequalities among the rural elderly. According to healthy life cycle theory, the older the elderly are, the lower the health level is. The health is influenced by economic factors gradually weakening and non-economic factors increasing. Therefore, according to the data in the table and the curves in the graph, it can be observed that as the age increases, the income-related health inequalities among the elderly gradually decrease.

### 3.3. Decomposition of the Factors Affecting Health Inequality Among the Rural Elderly in China

The Shapley decomposition method was used to analyze the factors contributing to health inequality among the elderly, and the contribution value and contribution rate of the four-dimensional factors were obtained, as shown in Table 6. This paper uses the RIF-I-OLS decomposition method to decompose the marginal effects of demographic characteristics, socioeconomic status (SES), health care access, and quality of later life on health inequality among the rural elderly, and the results are shown in Table 6.

#### 3.3.1. Demographic Characteristics and Health Inequality Among the Rural Elderly

The results of Shapley decomposition showed that demographic characteristics contributed 0.0054 to the health inequality index of the elderly, which accounted for 6.40% of the health inequality index. According to the regression function of health influencing factors, these contributions mainly come from age groups. From the results of RIF-I-OLS decomposition, we found that the marginal effects of age groups and marital status on health inequality are significant. The marginal effect coefficients of the 65–74 age group on WI and EI were 0.0664 and 0.0545, both with significant levels of 0.01. The marginal effect coefficients of the 75–84 age group on WI and EI were 0.0263 and 0.0198, and respectively, with significant levels of 0.01 and 0.05. The marginal effect coefficients of married on WI and EI were 0.0176 and 0.0156, respectively, with significant levels of 0.05 and 0.1.

#### 3.3.2. Socioeconomic Status (SES) and Health Inequality Among the Rural Elderly

The results of Shapley decomposition showed that socioeconomic status (SES) contributed 0.0130 to the health inequality index of the elderly, which accounted for 15.39% of the health inequality index. According to the regression function of health influencing factors, these contributions respectively come from income inequality, education, and region. From the results of RIF-I-OLS decomposition, we found that the marginal effects of the Gini coefficient of province, years of education, and western China on health inequality are significant. The marginal effect coefficients of the Gini coefficient on WI and EI were 0.5882 and 0.5440, respectively, both with significant levels of 0.01. The marginal effect coefficients of years of education on WI and EI were 0.0041 and 0.0037, respectively, both with significant levels of 0.01. The marginal effect coefficients of western China on WI and EI were −0.0338 and −0.0323, respectively, both with significant levels of 0.01.

#### 3.3.3. Health Care Access and Health Inequality Among the Rural Elderly

The results of Shapley decomposition showed that health care access contributed 0.0442 to the health inequality index of the elderly, which accounted for 52.41% of the health inequality index. According to the regression function of health influencing factors, these contributions respectively come from timely medical treatment, medical facilities, medical expenses, and the main medical payment channel. From the results of RIF-I-OLS decomposition, we found that the marginal effects of timely medical treatment, medical facilities, medical expenses, and insurance payment on health inequality are significant. The marginal effect coefficients of timely medical treatment on WI and EI were −0.0286 and −0.0284, respectively, both with significant levels of 0.01. The marginal effect coefficients of the number of medical institution beds per 1000 people on WI and EI were −0.0131 and −0.0116, respectively, both with significant levels of 0.1. The marginal effect coefficients of medical expenses on WI and EI were −0.5795 and −0.5206, respectively, both with significant levels of 0.01. The marginal effect coefficients of insurance payment on WI and EI were 0.0167 and 0.0153, respectively, both with significant levels of 0.1.

#### 3.3.4. Quality of Later Life and Health Inequality Among the Rural Elderly

The results of Shapley decomposition showed that quality of later life contributed 0.0218 to the health inequality index of the elderly, which accounted for 25.80% of the health inequality index. According to the regression function of health influencing factors, these contributions respectively come from living arrangements, pension, and physical exercise. From the results of RIF-I-OLS decomposition, we found that the marginal effects of living in nursing homes, pension, and physical exercise on health inequality are significant. The marginal effect coefficients of living in nursing homes on WI and EI were −0.1111 and −0.1031, respectively, both with significant levels of 0.01. The marginal effect coefficients of pension on WI and EI were 0.0612 and 0.0575, respectively, both with significant levels of 0.01. The marginal effect coefficients of physical exercise on WI and EI were 0.0257 and 0.0216, respectively, both with significant levels of 0.01.

## 4. Discussion

The measurement shows that the health of the rural elderly in China improved with social economic development and medical reform from 2002 to 2014. However, at the same time, we were surprised to find that the health level of the elderly in the younger age group has been declining steadily since 2008. This phenomenon implies that the incidence of chronic diseases is moving towards the younger elderly. This finding is similar to the results of Pishkar et al.’s research conclusion, whose research findings in terms of the elderly people of Iranshahr showed that the mean age of older people is 66.33 ± 7.7, the highest frequency belongs to the age group of 60 years, and the maximum age is 92 years. In total, 69.5% of the older people were in the age group of the young elderly (60–69 years old). The mean score of geriatric health promotion behaviors was 6.1 ± 1.87 in the range of 0–11 and 54.9% of them had a score of inappropriate health promotion behavior [23]. 

The measurement of health inequality shows that there is indeed pro-rich health inequality among the rural elderly, the health inequality of the younger age groups is more serious than that of the older age groups, and the former incidence of health inequality is higher. The changing trend of health inequality among the elderly in rural areas of China exhibits a structural change with different age groups. Health inequality in the group of 65–74 years old is higher than that in other groups, and the trend of change is fluctuating downward. Health inequality in the group of 75–84 years old is lower than that in the group of 65–74 years old, but higher than that in the other age groups. If there is a competitive relationship between age and SES when they have impacts on health inequality, we need to focus more on the health status of the elderly with low SES [24]. Fonta’s study on health inequality among the elderly in Ghana found the same conclusion: the poor suffer more health inequalities [25].

Demographic characteristics, such as age groups and marital status, are the most basic determinants of the health of the elderly. Health life cycle theory and life course theory are used to explain the impact of demographic characteristics on the health of the elderly [26,27]. Age should be considered as the most basic classification variable when analyzing the health inequality of the elderly. Health inequality is only meaningful when comparing the same age group, because there must be differences in the health status among the elderly of different ages. The rule applies to other countries, for example, Ghana. Fonta’s study showed that the odds of reporting poor health were 1.6 times and 2.5 times higher among the middle old and old-old, respectively. The marginal coefficients of the 65–74, 75–84, and 85–94 age groups for the health inequality index decrease in turn, and the significance is less pronounced, indicating that the characteristics of health inequality with age are in agreement with healthy life cycle theory. The data in Table 3 show that 75.55% of rural elderly in China were divorced, separated, widowed, or single when visited. The difference in healthy marital status is obvious. 

Health inequality related to SES is the most popular research topic in the field [28]. Like other studies, this paper analyses the impact of SES, including income inequality, education, and region, on health inequality of the elderly in rural China. The impact of income inequality on health is obvious. Current studies have basically reached a consensus on this. The higher one’s income, the better one’s health [29]. Education affects health outcomes through health behavior. There are differences in health behavior, so there are differences in health outcomes, which leads to health inequality being related to education. Higher education often leads to higher levels of health outcomes [30]. The health of the elderly often differs according to their living region. In addition, the World Health Organization’s 2016 Global Report on Ageing and Health points out that the health inequalities caused by social and economic resources will accumulate over a long period of time and eventually lead to differences in the internal ability and function of the elderly [1]. 

In particular, this paper explores the impact of health care access on the health inequality among the elderly in rural China. As expected, it can be concluded that the higher the health care access, the lower the health inequality of the elderly. In fact, medical accessibility can be analyzed from both suppliers and consumers [31]. Provider accessibility describes the supply capacity of medical service resources, including medical facilities. Demander accessibility describes the ability of consumers to pay, including medical expenses. China’s medical service system is special, and both big hospitals and good hospitals are public. There is a mismatch between the supplier and the demander in China’s medical market. There are many patients in tertiary hospitals, while few people visit primary hospitals. The uneven distribution of medical service resources has become an important factor, leading to health inequality of the elderly in rural China.

The quality of later life has a significant impact on health inequality among the elderly. Living in a nursing home weakens the health inequalities between low-income seniors and high-income seniors. When the elderly live in the same nursing home, the accommodation and accommodation arrangements are more unified. The medical care that the old people enjoy are basically the same. This situation weakens the health differences caused by socioeconomic factors to a certain extent. The RIF-I-OLS decomposition coefficient of the pension insurance is significantly positive at the statistical level of 0.01, which means that pension insurance is more beneficial to the health of high-income seniors. During the investigation period, most of the rural elderly did not have pension insurance. The elderly who had been employed in formal departments, units, and enterprises may receive retirement wages. However, most elderly people who mainly rely on agriculture do not have corresponding pension insurance. Their pension funds come from the income of agricultural production activities, children, or their own work. This unbalanced situation leads to differences in health investment between high-income seniors and low-income seniors and expands the health inequalities among the elderly people in rural areas [32]. The decomposition coefficient of the current physical exercise is significantly positive at the statistical level of 0.01, indicating that the current physical exercise is more beneficial to the health of the elderly in high-income families.

## 5. Conclusions

The measurement shows that the health of the rural elderly in China improved with social economic development and medical reform from 2002 to 2014. However, at the same time, we were surprised to find that the health level of the elderly in the younger age group has been declining steadily since 2008. This phenomenon implies that the incidence of chronic diseases is moving towards the younger elderly. The measurement of health inequality shows that there is indeed pro-rich health inequality among the rural elderly, the health inequality of the younger age groups is more serious than that of the older age groups, and the former incidence of health inequality is higher. The health inequality in the group of 65–74 years old is higher than that in other groups, and the trend of change fluctuated downward from 2002 to 2014. The health inequality in the group of 75–84 years old is lower than that in the group of 65–74 years old, but higher than that in the other age groups. The results of Shapley decomposition show that demographic characteristics, socioeconomic status (SES), health care access, and quality of later life contributed 0.0054, 0.0130, 0.0442, and 0.0218 to the health inequality index of the elderly, which accounted for 6.40%, 15.39 %, 52.41%, and 25.80 % of health inequality index, respectively. From the results of RIF-I-OLS decomposition, this paper has analyzed detailed factors’ marginal effects on health inequality from four dimensions, which indicates that the health inequality among the elderly in rural China was mainly caused by the disparity of income, medical expenses, and living arrangement.

## Figures and Tables

**Table 1:** Chinese Longitudinal Healthy Longevity Survey (CLHLS) data set sample content structure (2002–2014).

Year	Sample of Survey Year	Continuous Survey Sample	Survey Year Rural Sample	Rural Sample for Continuous Survey
2002	16,064	—	8670	—
2005	15,638	8175 (2002–2005)	8658	4337
2008	16,954	4191 (2002–2008)	10,293	1897
2011	9765	2513 (2002–2011)	5145	737
2014	7192	1681 (2002–2014)	3980	332

Note: According to the introduction of CLHLS data.

**Table 2:** Quality of Well-Being (QWB) scale items weights and corresponding number of the CLHLS questionnaire.

	Content	Weight	Questionnaire No.
	**Mobility Scale (MOB)**		
4	Did not drive a car, health related; did not ride in a car as usual for age (younger than 15 yr), health related, and/or did not use public transportation, health related; or had or would have used more help than usual for age to use public transportation, health related.	−0.062	E4, E14
	**Physical Activity Scale (PAC)**		
3	In wheelchair, moved or controlled movement of wheelchair without help from someone else; or had trouble or did not try to lift, stoop, bend over, or use stairs or inclines, health related; and/or limped, used a cane, crutches, or walker, health related; and/or had any other physical limitation in walking, or did not try to walk as far or as fast as others the same age are able, health related.	−0.060	G9, G11, E11–E13
	**Social Activity Scale (SAC)**		
4	Limited in other (e.g., recreational) role activity, health related.	−0.061	E6–E8
3	Limited in major (primary) role activity, health related.	−0.061	E2–E3
2	Performed no major role activity, health related, but did perform self-care activities.	−0.061	E3
1	Performed no major role activity, health related, and did not perform, or had more help than usual in performance of one or more self-care activities, health related.	−0.106	E1–E3, E6, E9–E10
	**Symptom/Problem Complexes (CPX)**		
5	Trouble learning, remembering, or thinking clearly.	−0.340	C54–C55, G15O1
6	Any combination of one or more hands, feet, arms, or legs either missing, deformed (crooked), paralyzed (unable to move), or broken—includes wearing artificial limbs or braces.	−0.333	C55
8	Pain, burning, bleeding, itching, or other difficulty with rectum, bowel movements, or urination (passing water).	−0.292	E5, G15B1, G15S1
12	Spells of feeling upset, being depressed, or of crying.	−0.257	B23–B24, B26
18	Pain in ear, tooth, jaw, throat, lips, tongue; several missing or crooked permanent teeth—includes wearing bridges or false teeth; stuffy, runny nose; or any trouble hearing-includes wearing a hearing aid.	−0.170	C55, G22, H1

Note: Compilation of results with reference to Kaplan and Anderson’s research [15].

**Table 3:** Descriptive statistics of the main variables.

Variables	Description	Mean ± SD or *n* (%)
Health Status	QWB Score	0.6276 ± 0.1500
**Demographic characteristics**		
**Gender**	Male	7923 (40.96)
	Female	11,418 (59.04)
**Age groups**	Age group1 (65–74)	2149 (11.11)
	Age group2 (75–84)	3255 (16.83)
	Age group3 (85–94)	6980 (36.09)
	Age group4 (95+) ^a^	6957 (35.97)
**Marital status**	Married	4728 (24.45)
	Divorced, separated, widowed, or single	14,613 (75.55)
**Socioeconomic status (SES)**		
**Income inequality**	Gini coefficient of province	0.3677 ± 0.0749
**Education**	Years of education	1.3810 ± 2.6982
**Region**	Eastern China ^a^	8001 (41.37)
	Central China	6313 (32.64)
	Western China	5027 (25.99)
**Health care access**		
**Timely medical treatment**	Yes	17,037 (88.09)
	No	2304 (11.91)
**Medical facilities**	Number of medical institution beds per 1000 people	1.0190 ± 0.4847
**Medical expenses**	Total medical expenses last year (yuan)	1,343.0770 ± 4,827.2570
**Main medical payment channel**	Medical insurance	3294 (17.03)
	Other	16,047 (82.97)
**Quality of later life**		
**Living arrangements**	Living in nursing homes	346 (1.79)
	Not living in nursing homes	18,995 (98.21)
**Pension**	Yes	996 (5.15)
	No	18,345 (94.85)
**Physical exercise**	Yes	3513 (18.16)
	No	15,828 (81.84)

Note: ^a^ represents the reference group; Gini coefficients of province were embedded from macro data to data sets according to the province no.; Consumer Price Index (CPI) is deducted last year’s total medical expenses.

**Table 4:** QWB scores distribution of the elderly in different age groups from 2002 to 2014.

	2002	2005	2008	2011	2014
65–74 years old	0.7730	0.7772	0.7832	0.7810	0.7770
75–84 years old	0.7128	0.7157	0.7197	0.7233	0.7276
85–94 years old	0.6357	0.6478	0.6396	0.6433	0.6600
Above 95 years old	0.5639	0.5747	0.5627	0.5725	0.5868

**Table 5:** Distribution of Wagstaff index (WI) and Erreygers index (EI) in different age groups from 2002 to 2014.

	2002		2005		2008		2011		2014	
	WI	EI	WI	EI	WI	EI	WI	EI	WI	EI
65–74 years old	0.0870	0.0826	0.0595	0.0567	0.1060	0.1030	0.0734	0.0716	0.0379	0.0360
75–84 years old	0.0557	0.0534	0.0818	0.0790	0.0474	0.0455	0.0063	0.0061	0.0784	0.0763
85–94 years old	0.0517	0.0516	0.0322	0.0322	0.0352	0.0351	0.0031	0.0031	0.0035	0.0035
Above 95 years old	0.0379	0.0377	0.0575	0.0574	0.0124	0.0123	0.0232	0.0231	0.0279	0.0276

**Table 6:** Shapley decomposition and recentered influence function-index-ordinary least squares (RIF-I-OLS) decomposition of health inequality among the elderly in rural China.

Dimensions	Variables	Shapley Decomposition		RIF-I-OLS Decomposition	
		Health (QWB)	Contribution (rate, %)	RifWI	RifEI
**Demographic characteristics**	Male	0.0083	0.0054 (6.40%)	−0.0058	−0.0064
		(0.4263)		(−0.8537)	(−1.0205)
	Age group1 (65–74)	0.0293		0.0664 ***	0.0545 ***
		(0.8888)		(4.9352)	(4.3412)
	Age group2 (75–84)	−0.1525 ***		0.0263 ***	0.0198 **
		(-5.6868)		(2.6841)	(2.1718)
	Age group3 (85–94)	−0.0962 ***		0.0084	0.0057
		(−4.6733)		(1.2847)	(0.9398)
	Married	−0.0264		0.0176 **	0.0156 *
		(−1.1330)		(2.0328)	(1.9386)
**Socioeconomic status (SES)**	Gini coefficient	−1.2790 ***	0.0130 (15.39%)	0.5882 ***	0.5440 ***
		(−4.0629)		(5.1053)	(5.0656)
	Years of education	0.0130 ***		0.0041 ***	0.0037 ***
		(3.5411)		(2.8928)	(2.8011)
	Central China	−0.1482 ***		0.0091	0.0084
		(−7.0585)		(1.2016)	(1.1894)
	Western China	−0.1238 ***		−0.0338 ***	−0.0323 ***
		(−4.6590)		(−3.4009)	(−3.4806)
**Health care access**	Timely medical treatment	0.4802 ***	0.0442 (52.41%)	−0.0286 ***	−0.0284 ***
		(16.6442)		(-3.1046)	(−3.2866)
	Medical facilities	0.0679 ***		−0.0131 *	−0.0116 *
		(3.0847)		(−1.7954)	(−1.7222)
	Medical expenses	−4.3496 ***		−0.5795 ***	−0.5206 ***
		(−9.0786)		(−5.6915)	(−5.5188)
	Insurance payment	0.0438 *		0.0167 *	0.0153 *
		(1.9154)		(1.7990)	(1.7748)
**Quality of later life**	Living in nursing homes	−0.0826	0.0218 (25.80%)	−0.1111 ***	−0.1031 ***
		(−1.0760)		(−3.4715)	(−3.4544)
	Pension	0.0745 *		0.0612 ***	0.0575 ***
		(1.9425)		(3.8966)	(3.9514)
	Physical exercise	0.3221 ***		0.0257 ***	0.0216 ***
		(15.4757)		(3.1152)	(2.8047)
	Constant	3.5439 ***	—	−0.1451 ***	−0.1295 ***
		(26.8070)	—	(−3.0643)	(−2.9365)
	Time fixed effect	Controlled	—	Controlled	Controlled
**Total Contribution**	—	—	0.0844 (100%)	—	—

Note: Age group4 (95+) and eastern China are the reference groups; *, **, and *** indicate significant levels of 0.1, 0.05, and 0.01, respectively, with the values in parentheses being standard errors of robustness.
